# Laparoscopic excision of a large prostatic utricle in an infant with 46,xx Sry-negative differences of sex development: a case report

**DOI:** 10.1093/jscr/rjag664

**Published:** 2026-07-31

**Authors:** Christos Costa, Arindam Dastidar, Ashok Rijhwani, Fiona Ryan, Debbie Shears, Mary Owen, Subhasis Chakraborty, Foteini Moniati, Kristina Dzhuma

**Affiliations:** Department of Paediatric Urology, John Radcliffe Hospital, Oxford University Hospitals NHS Foundation Trust, Oxford, United Kingdom; Department of Paediatric Urology, John Radcliffe Hospital, Oxford University Hospitals NHS Foundation Trust, Oxford, United Kingdom; Department of Paediatric Urology, John Radcliffe Hospital, Oxford University Hospitals NHS Foundation Trust, Oxford, United Kingdom; Department of Paediatric Endocrinology, John Radcliffe Hospital, Oxford University Hospitals NHS Foundation Trust, Oxford, United Kingdom; Oxford Centre of Genomic Medicine, John Radcliffe Hospital, Oxford University Hospitals NHS Foundation Trust, Oxford, United Kingdom; Clinical Psychology Department, Department of Diabetes and Endocrinology, John Radcliffe Hospital, Oxford University Hospitals NHS Foundation Trust, Oxford, United Kingdom; Department of Paediatric Radiology, John Radcliffe Hospital, Oxford University Hospitals NHS Foundation Trust, Oxford, United Kingdom; Department of Inflammation and Ageing, University of Birmingham, Birmingham, United Kingdom; Department of Paediatric Urology, John Radcliffe Hospital, Oxford University Hospitals NHS Foundation Trust, Oxford, United Kingdom

**Keywords:** prostatic utricle, hypospadias, differences of sex development, laparoscopy, infant, micturating cystourethrogram

## Abstract

Prostatic utricles are commonly associated with hypospadias and differences of sex development (DSD) but are rarely large. We report a 6-month-old infant with 46,XX SRY-negative DSD and distal hypospadias, found to have a large pelvic cystic lesion measuring up to 6.7 cm. Serial imaging suggested a Müllerian duct remnant, while micturating cystourethrogram (MCUG) confirmed communication with the posterior urethra and a markedly enlarged prostatic utricle consistent with Ikoma grade III. The patient underwent cystoscopy-guided catheterization and laparoscopic excision. Complete removal was achieved with preservation of adjacent structures and an uncomplicated recovery. This case highlights the diagnostic value of MCUG and demonstrates that minimally invasive excision provides excellent visualization and enables safe excision in infancy.

## Introduction

Prostatic utricles are Müllerian duct remnants commonly associated with proximal hypospadias and differences of sex development (DSD) [1–3]. While most are small and asymptomatic, enlarged utricles may present as pelvic cystic lesions and can be associated with urinary tract infection, voiding dysfunction, or post-void dribbling [3, 4].

Ikoma *et al*. proposed a widely used grading system based on utricle size relative to the bladder neck, ranging from grade 0 (not extending beyond the verumontanum) to grade III (extending well above the bladder neck) [5].

Accurate diagnosis can be challenging, as these lesions may mimic other midline pelvic cysts, including Müllerian duct remnants or duplication cysts [6]. Confirmation of communication with the posterior urethra is essential, with micturating cystourethrogram (MCUG) playing a key diagnostic role [6].

Management is guided by lesion size, symptoms, and anatomical considerations. Larger utricles may require surgical excision, particularly when they are likely to interfere with urinary function or future reconstructive procedures. Minimally invasive approaches offer improved visualizations within the deep pelvis and facilitate precise dissection of the utricle and its neck [7].

We present a case of a large prostatic utricle in an infant with DSD, highlighting the diagnostic pathway and key operative considerations in its management.

## Case presentation

A 6-month-old phenotypic male infant was referred to the paediatric urology service for evaluation of atypical genitalia and an incidentally identified pelvic cystic lesion. Initial assessment had been undertaken within a DSD multidisciplinary framework, confirming a 46,XX chromosome complement with no evidence of the SRY gene. Further genetic testing, including SNP microarray and a targeted DSD gene panel, identified no causative variants. Endocrine evaluation demonstrated a normal testosterone response to hCG stimulation (baseline 4.1, peak 38.3 nmol/L), normal gonadotropin response on LHRH testing (peak LH 16.2 IU/L, FSH 7.9 IU/L), and elevated anti-Müllerian hormone consistent with functioning testicular tissue. A urine steroid profile excluded congenital adrenal hyperplasia. The distinction between 46,XX testicular DSD and ovotesticular DSD remains pending, as gonadal biopsy is planned at a later stage alongside future reconstructive surgery. From a surgical perspective, the principal concern was the presence of a large pelvic cyst in the context of complex urogenital anatomy.

On clinical examination of external genitalia (Fig. 1A and B) a small phallus with meatus located at the upper third of the shaft with associated chordee was identified, consistent with distal hypospadias. Labioscrotal folds were well developed and fused, resulting in the appearance of a well-developed bifid scrotum with penoscrotal transposition. Both gonads were palpable, with the right located at the scrotal neck and the left within the scrotum, each of normal size and consistency.

**Figure 1:**
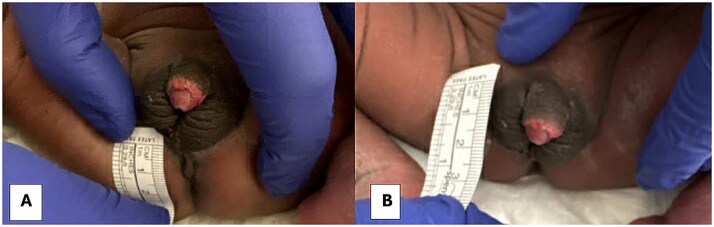
(A, B) Clinical photographs of the external genitalia demonstrating a small phallus with the urethral meatus located at the upper third of the penile shaft, associated with chordee, consistent with distal hypospadias.

Ultrasonography (US) performed on Day 2 of life demonstrated bilateral homogeneous gonads within the inguinal regions, with no identifiable internal female reproductive structures. A well-defined, thin-walled cystic lesion was identified posterior to the bladder measuring ~51 × 32 × 26 mm, containing dependent echogenic debris (Fig. 2A and B). A repeat US 2 months later confirmed persistence of the lesion, measuring ~4.1 × 5.3 × 3.0 cm, with interval resolution of internal debris. A possible communication with the lower urinary tract was suspected at the caudal aspect of the cyst, although this could not be definitively demonstrated (Fig. 3A and B). Normal upper urinary tract was noted. The size, persistence, and anatomical location of the lesion raised suspicion for a prostatic utricle.

**Figure 2:**
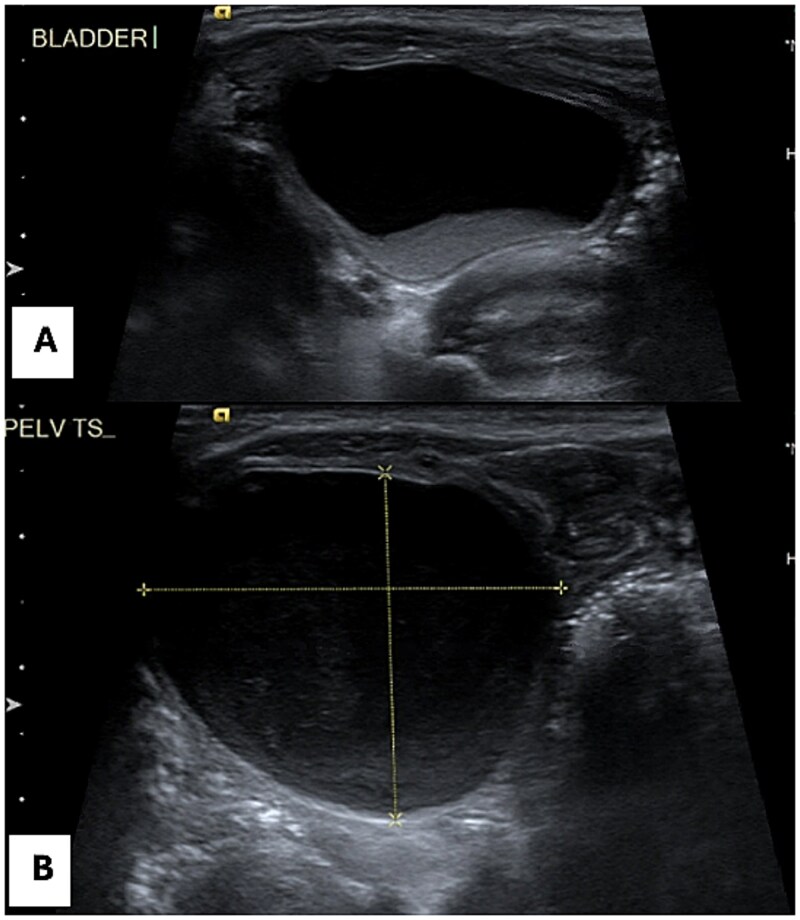
(A, B) Pelvic ultrasonography demonstrating a thin-walled unilocular cystic lesion posterior to the bladder, measuring ~ 51 ×32 × 26 mm, with dependent echogenic fluid–fluid level.

**Figure 3:**
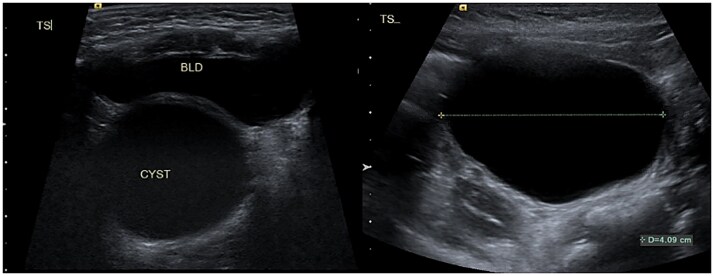
Repeat pelvic ultrasonography demonstrating a persistent thin-walled cystic lesion posterior to the bladder, measuring ~4.1 × 5.3 × 3.0 cm, now containing anechoic fluid with resolution of prior internal debris.

Further anatomical characterization with magnetic resonance imaging (MRI) demonstrated a large, elongated midline cystic structure located between the posterior bladder and anterior rectum, measuring ~6.7 × 3.0 × 3.2 cm. The lesion contained dependent layered internal signal and tapered caudally toward the expected course of the proximal urethra, although direct communication could not be visualized (Fig. 4). These findings were consistent with a Müllerian duct remnant, most likely a prostatic utricle.

**Figure 4:**
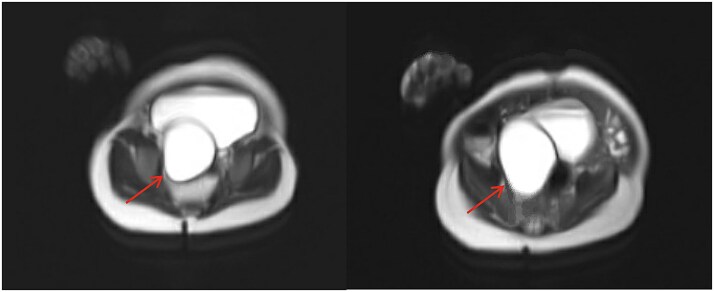
Pelvic MRI demonstrating a large midline cystic structure between the bladder and rectum, measuring ~6.7 × 3.0 × 3.2 cm, with dependent fluid–fluid levels and caudal tapering toward the proximal urethra. The arrow indicates the prostatic utricle.

An MCUG was subsequently performed. This demonstrated a normal bladder and urethra, with contrast passing during voiding from the prostatic urethra into a posterior midline cystic structure, confirming communication with a markedly enlarged prostatic utricle, consistent with Ikoma grade III (Fig. 5). No vesicoureteric reflux was identified. This represented the key diagnostic step and established the indication for surgical management.

**Figure 5:**
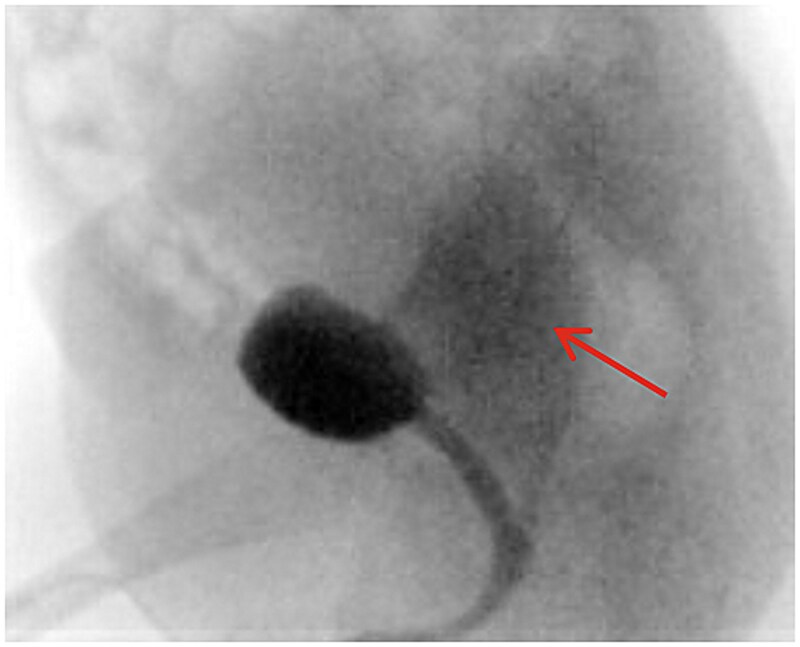
Micturating cystourethrogram demonstrating a normal bladder and urethra, with contrast passing during voiding from the prostatic urethra into a posterior midline structure, confirming communication with a prostatic utricle. No vesicoureteric reflux is seen. The arrow indicates the prostatic utricle.

Following confirmation of the diagnosis, the case was discussed at the paediatric urology multidisciplinary team meeting. Surgical excision was recommended in view of ongoing urinary reflux into the utricle, significant size of the cyst and presence of layering debris on the imaging suggestive of increased risk for urinary tract infection and potential haematuria in the future.

The risks of surgery were outlined to the parents, including potential injury to adjacent structures such as the urethra, bladder, and vas deferens, as well as the possibility of incomplete excision due to the close anatomical relationship of the utricle to surrounding structures. A minimally invasive approach was proposed for prostatic utricle excision with ongoing antibiotic prophylaxis with trimethoprim in the meantime. The decision was made for the hypospadias to be dealt with at a later stage due to the small size of the glans and the phallus, with additionally possible need for gonadal biopsy to assess for ovarian tissue in view of the underlying SRY-negative DSD.

The patient underwent diagnostic cystoscopy followed by laparoscopic excision. Examination under anaesthesia confirmed distal hypospadias with the meatus just below the corona and both gonads palpable.

An 8 Fr paediatric cystoscope was introduced following meatal dilation to 9 Fr. The verumontanum was identified with a midline opening consistent with the utricle orifice. A guidewire could not be advanced into the utricle; an 8 Fr Foley catheter was placed into the bladder as an intraoperative landmark.

Pneumoperitoneum was established via a transumbilical 5 mm port at 8–10 mmHg, with two additional 5 mm working ports. Laparoscopic inspection confirmed a large retrovesical cyst with no additional Müllerian structures. The lesion was mobilized using hook diathermy, scissors, and harmonic dissection, with a hitch stitch to improve pelvic exposure. The utricle neck was identified, secured with two Vicryl endoloops, and divided 5 mm above the ligation point. The specimen was retrieved via the umbilical port (Fig. 6 and Video).

**Figure 6:**
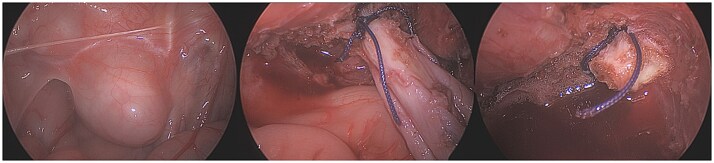
Intraoperative images demonstrating the sequential steps of prostatic utricle excision. From left to right: (1) identification of the prostatic utricle, (2) meticulous dissection and ligation of the utricle at its neck, and (3) complete resection of the prostatic utricle.

Histopathological analysis demonstrated fibrovascular tissue lined predominantly by squamous mucosa with focal urothelial epithelium. No prostatic glandular epithelium, significant inflammation, or morphological evidence of malignancy was identified, consistent with the diagnosis of prostatic utricle.

The postoperative course was uncomplicated. The patient tolerated feeds, maintained adequate urine output, and showed no evidence of infection or urinary complications. He was discharged with a urinary catheter in situ using a double nappy technique. His urethral catheter was removed 2 weeks later uneventfully and the patient is passing urine without difficulty.

Trimethoprim prophylaxis was discontinued following catheter removal. At 3 months postoperatively, the patient remains well with no urinary tract infections, no recurrent urinary symptoms, and no clinical evidence of recurrence. The timing of hypospadias repair will be determined at the next clinic review, alongside consideration of gonadal biopsy to establish the definitive histological diagnosis.

## Discussion

Prostatic utricles are Müllerian duct remnants frequently associated with proximal hypospadias and DSD [1, 2]. While their presence is relatively common in this cohort, most are small and clinically insignificant [3]. In contrast, enlarged utricles may present as pelvic cystic lesions and pose both diagnostic and surgical challenges, particularly in infants with complex urogenital anatomy [2, 4]. The present case is notable for the size of the lesion and its clear communication with the posterior urethra, rendering it clinically relevant from a paediatric urology perspective.

This patient carries a diagnosis of 46, XX SRY-negative DSD, a rare condition in which testicular differentiation occurs despite the absence of SRY. Possible aetiological mechanisms include gain-of-function rearrangements involving downstream testis-determining genes such as SOX9 or SOX3, though no causative variant was identified on routine testing [11, 12]. Notably, Wei *et al*. reported a prostatic utricle in an SRY-negative 46,XX individual harbouring an SOX3 duplication, underscoring the association between disruption of testis-determining pathways and the persistence of Müllerian remnants in this population [13]. In contrast to that report, which described a 5-year-old child, the present case is distinguished by the large size of the lesion (Ikoma grade III) presenting in infancy and by its successful laparoscopic management within this rare DSD context. Endocrine evaluation confirmed functioning testicular tissue; however, the formal distinction between testicular and ovotesticular DSD awaits gonadal histology. Despite this diagnostic uncertainty, the surgical considerations were driven primarily by anatomical findings rather than genetic background. The presence of distal hypospadias combined with a large midline pelvic cyst raised early suspicion for a prostatic utricle. It is well recognized that the incidence and size of utricles correlate with the severity of hypospadias [1, 2]. This case reinforces that management decisions in DSD should be guided by functional anatomy and anticipated reconstructive requirements rather than karyotype alone [6, 7].

Establishing the diagnosis remains a key challenge. Ultrasonography is typically the first-line investigation and is useful in identifying cystic pelvic structures, but it does not reliably demonstrate communication with the urethra [8]. In this case, serial ultrasound confirmed persistence of the lesion but could not definitively characterize its origin. MRI provided further anatomical detail but similarly did not confirm communication. The MCUG proved to be the decisive investigation, demonstrating contrast passage from the prostatic urethra into the cystic structure. This finding established the diagnosis and directly informed operative planning.

Cystoscopy plays an important complementary role in both diagnosis and operative strategy. Direct visualization of the utricle orifice at the verumontanum confirms the diagnosis and allows catheterization of the utricle. In this case, catheter placement into the utricle was not achieved; however, catheterization of the bladder ensured the urethra remained intact during the dissection and provided a reliable intraoperative landmark, facilitating identification of the lesion within a confined and anatomically complex pelvic space [3, 4]. This combined endoscopic and laparoscopic approach enhances anatomical orientation and reduces the risk of incomplete excision or injury to adjacent structures [9].

The decision to proceed with surgical excision was based on lesion size, demonstrated urethral communication, and anticipated impact on future hypospadias reconstruction. Larger utricles are associated with urinary stasis, infection, and voiding dysfunction, and may complicate subsequent reconstructive procedures [3, 4]. Addressing the utricle prior to definitive urethral repair allows optimization of lower urinary tract anatomy and may reduce postoperative complications [6]. In this context, early intervention was considered appropriate.

A range of surgical techniques has been described, including endoscopic, open, laparoscopic, and robotic approaches. Endoscopic techniques are less invasive but are associated with higher recurrence rates due to incomplete excision [3, 4]. Open approaches provide direct access but are limited by restricted visualization within the deep pelvis and increased morbidity, particularly in infants [9]. Minimally invasive approaches allow improved visualization and facilitate precise dissection of the utricle and its neck [9–11].

From a technical perspective, several operative principles were critical in this case. First, preoperative confirmation of urethral communication ensured accurate diagnosis and informed surgical planning. Second, meticulous identification and controlled division of the utricle neck were essential to minimize the risk of recurrence while avoiding urethral injury. Adjuncts such as traction using a hitch stitch improved exposure within the confined pelvic space and facilitated safe dissection. These principles are reproducible and particularly relevant in infants, where operative working space is limited.

Incomplete excision remains an important consideration. Residual utricle tissue may lead to persistent symptoms or recurrence, particularly when less invasive techniques are used [3, 4, 10]. This supports a definitive surgical approach in larger lesions, where complete excision can be achieved safely with appropriate technique.

The timing of intervention in infants with DSD remains an area of ongoing discussion. While delayed management may be appropriate in selected cases, early excision of large utricles may reduce the risk of complications and simplify subsequent reconstructive procedures [6, 7]. In this case, early surgical management was guided by lesion size, anatomical considerations, and anticipated reconstructive needs.

Overall, this case highlights the importance of a structured diagnostic pathway and a deliberate surgical approach in the management of large prostatic utricles. Accurate diagnosis requires confirmation of urethral communication, and safe excision depends on careful operative planning, clear identification of the utricle neck, and preservation of surrounding structures. A limitation of this report is the relatively short postoperative follow-up of 3 months; longer-term surveillance is required to confirm the absence of recurrence and to assess functional urinary outcomes.

## Conclusion

Large prostatic utricles in infants with differences of sex development present both diagnostic and technical surgical challenges. Confirmation of communication with the posterior urethra is essential, with micturating cystourethrogram playing a decisive role when initial imaging is inconclusive. Laparoscopic excision allows safe and effective removal, providing excellent visualization within the deep pelvis and facilitating precise identification of the utricle and its neck. Early intervention in selected cases may optimize lower urinary tract anatomy and support subsequent reconstructive procedures.

## Learning points

- Large prostatic utricles should be considered in infants with hypospadias presenting with pelvic cystic lesions.
- Demonstration of communication with the posterior urethra is essential for diagnosis, with MCUG being the most informative investigation.
- Cystoscopy is valuable for confirming the diagnosis and facilitating intraoperative orientation through catheter placement.
- Surgical indication should be based on size, symptoms, and potential impact on future reconstructive procedures.
- Careful identification of the utricle neck and preservation of adjacent structures are key operative principles to ensure safe and complete excision.

## Supplementary Material

Prostatic_Utricle_Video_Final_rjag664
